# *L. plantarum, L. salivarius,* and *L. lactis* Attenuate Th2 Responses and Increase Treg Frequencies in Healthy Mice in a Strain Dependent Manner

**DOI:** 10.1371/journal.pone.0047244

**Published:** 2012-10-09

**Authors:** Maaike J. Smelt, Bart J. de Haan, Peter A. Bron, Iris van Swam, Marjolein Meijerink, Jerry M. Wells, Marijke M. Faas, Paul de Vos

**Affiliations:** 1 Top Institute Food and Nutrition, Wageningen, The Netherlands; 2 Department of Pathology and Medical Biology, University Medical Center Groningen and University of Groningen, Groningen, The Netherlands; 3 NIZO Food Research, Ede, The Netherlands; 4 Department of Host-Microbe Interactomics, Wageningen University, Wageningen, The Netherlands; 5 Kluyver Centre for Fermentation and Genomics, Delft, The Netherlands; Instituto Butantan, Brazil

## Abstract

Many studies on probiotics are aimed at restoring immune homeostasis in patients to prevent disease recurrence or reduce immune-mediated pathology. Of equal interest is the use of probiotics in sub-clinical situations, which are characterized by reduced immune function or low-grade inflammation, with an increased risk of infection or disease as a consequence. Most mechanistic studies focus on the use of probiotics in experimental disease models, which may not be informative for these sub-clinical conditions. To gain better understanding of the effects in the healthy situation, we investigated the immunomodulatory effects of two *Lactobacillus* probiotic strains, i.e. *L. plantarum* WCFS1 and *L. salivarius* UCC118, and a non-probiotic *lactococcus* strain, i.e. *L. lactis* MG1363, in healthy mice. We studied the effect of these bacteria on the systemic adaptive immune system after 5 days of administration. Only *L. plantarum* induced an increase in regulatory CD103^+^ DC and regulatory T cell frequencies in the spleen. However, all three bacterial strains, including *L. lactis,* reduced specific splenic T helper cell cytokine responses after *ex vivo* restimulation. The effect on IFN-γ, IL5, IL10, and IL17 production by CD4^+^ and CD8^+^ T cells was dependent on the strain administered. A shared observation was that all three bacterial strains reduced T helper 2 cell frequencies. We demonstrate that systemic immunomodulation is not only observed after treatment with probiotic organisms, but also after treatment with non-probiotic bacteria. Our data demonstrate that in healthy mice, lactobacilli can balance T cell immunity in favor of a more regulatory status, via both regulatory T cell dependent and independent mechanisms in a strain dependent manner.

## Introduction

The intestinal microbiota is largely mutualistic in nature and important for human health. Apart from its well-established role in nutrition, it is important in the development of the immune system and the maintenance of homeostasis of tolerance and immunity [1]. For instance, aberrant microbial colonization results in abnormal development of secondary lymphoid organs, reduced peripheral CD4^+^ T cell frequencies, skewing towards a T helper-2 immune phenotype, absence of T and B cells in the gut-associated lymphoid tissue, and reduced immunoglobulin levels [2]–[4]. Further, an aberrant intestinal microbiota is not only associated with the risk of infection and the development of intestinal immune disorders, but also with immune disorders beyond the intestine, such as allergic skin- and respiratory disease [5]–[7], and autoimmune diseases such as rheumatoid arthritis [8], [9] and diabetes type I [10].

Administration of indigenous, non-pathogenic probiotic bacteria is a promising strategy to improve immune homeostasis and to maintain host health. Probiotics may improve host health by normalizing existing undesired immune responses, as is the case in allergy or autoimmune disease [11], [12]. These beneficial effects have been described in both diseased humans [13]–[15], as well as in experimental disease models [16]–[19]. Besides the beneficial effects in disease, probiotics may benefit persons who are not receiving medical treatment, but have an increased risk of infection or disease due to the deterioration or inflammatory status of their immune system. This applies not only for age-related changes in immune function [20], but also for individuals with a genetic predisposition [21], obesity [22], or malnutrition- [23], stress- or lifestyle-induced declining immune function [24]. Although the beneficial effects of probiotics in non-diseased subjects have been described in experimental vaccination trails [25]–[27] and infection studies [25], [28]–[35], the immunomodulatory mechanisms behind these effects remain poorly understood.

Knowledge of how different probiotic strains can affect the immune system in the absence of disease, will gain mechanistic insights and help clarify the magnitude of their effects on the immune system, the strain dependency of these effects, their safety, and potential applications in maintaining or improving immune homeostasis. Surprisingly, the number of studies investigating the immunomodulatory effects of probiotics in non-diseased subjects is limited [36]–[38]. Most studies have focused on disease models [39], with a strong perturbation of immune homeostasis and often skewing to a specific T helper cell response [39], [40]. Moreover, some disease models have a severely compromised intestinal barrier, which could alter the accessibility of the probiotics to the immune cells and the lymphoid tissues [41]. Therefore, studies in the disease state may not reflect and predict the immunomodulatory effects of probiotics in healthy individuals or persons with sub-optimal immune health. To address this gap in our understanding, we have investigated the immunomodulatory effects of probiotic bacteria in healthy, non-diseased mice.

The effects of orally administered *L. plantarum* WCFS1, *L. salivarius* UCC118, and *L. lactis* MG1363 on systemic T and dendritic cell populations and responses were investigated. Both *L. plantarum* WCFS1 and *L. salivarius* UCC118 are probiotic strains [42], [43], whereas *L. lactis* MG1363 is not associated with probiotic effects [44], [45]. The bacteria were administered over 5 days, which is the period required for mice to develop an adaptive immune response [44], [45]. In this study, we demonstrated strain dependent effects of the bacteria on dendritic cells *in vitro* and *in vivo* and specific T cell responses *in vivo* and *ex vivo*.

## Materials and Methods

### Bacterial Strains and Growth Conditions

*L. plantarum* WCFS1 [46] and *L. salivarius* UCC118 [47] were cultured at 37°C in Man Rogosa and Sharpe (MRS) broth. *L. lactis* MG1363 [48] was cultured at 30°C in M17 broth containing 0.5% glucose. All bacterial cultures were grown overnight until the stationary phase of growth. Subsequently, the cultures were diluted 1∶1000 in fresh medium and cultured for a second night to allow optimal growth. The optical density at 600 nm was measured and the number of colony forming units (CFU) was calculated based on standard growth curves. For all cultured bacterial strains, an OD_600_-value of 1 corresponds to 1–2×10^9^ CFU/mL, which was confirmed by plating serial dilutions on MRS or M17 agar plates (data not shown). To avoid bacterial manipulation and cell death, extensive washing and centrifuging was avoided. After overnight culture, a small portion of bacteria was diluted in fresh, sterile MRS broth and immediately administered to the animals. The mice received either sterile MRS broth or 1–2×10^8^ CFU bacteria in 200 µL MRS via intragastric gavage, daily.

### In vitro Culture and Stimulation of Murine Dendritic Cells

Bone marrow cells were isolated and cultured as described by Lutz *et al* [49], with minor modifications. Briefly, femora and tibiae from female 6 weeks old Balb/c mice (Charles River Breeding Laboratories, Protagem MI), were removed and stripped of muscles and tendons. After soaking the bones in 70% ethanol and rinsing in PBS, bones were carefully crushed with a mortar to release the bone marrow cells. Cells were filtered using Steriflip filtration and washed with RPMI medium. Bone marrow cells (2–4×10^7^) were seeded into Petri dishes in 10 ml RPMI 1640 Glutamax (Sigma–Aldrich, St. Louis, MO, USA) containing 10% (v/v) heat-inactivated fetal calf serum supplemented with penicillin (100 U/ml), streptomycin (100 µg/ml), 50 µm β-mercaptoethanol, and 20 ng/ml murine GM-CSF (R&D systems). The cells were incubated for 8 days at 37°C in 5% CO_2_ humidified atmosphere. On day 3, 10 ml was removed and replaced with complete medium. On day 5, 5 ml fresh medium was added. On day 7, immature dendritic cells were collected and seeded in a 24 wells plate at 5×10^5^ cells/well. On day 8, the cells were either left unstimulated or stimulated with *L. plantarum* WCFS1, *L. salivarius* UCC118, *L. lactis* MG1363 (1∶10 cell to bacteria ratio), or LPS (1 µg/mL). After 24 hours the concentration of IL10 and TNFα was determined in the culture supernatants using cytometric bead array (BD Biosciences). The cells were stained for the dendritic cell marker CD11c and the activation markers CD40 and CD86, or appropriate isotype controls (BD Biosciences).

### Animals

Wild-type male Balb/c mice were purchased from Harlan (Harlan, Horst, The Netherlands). The animals were fed standard chow and water *ad libitum*. All animal experiments were performed after receiving approval of the institutional Animal Care Committee of the Groningen University. All animals received animal care in compliance with the Dutch law on Experimental Animal Care. The n-values were based on a mandatory power analysis. The values were 6 mice per experimental group, based on a type I error of 5% and a type II error of 10%.

To study the effect of lactobacilli on the systemic immune system, three bacterial strains (*L. lactis* MG1363, *L. salivarius* UCC118, and *L. plantarum* WCFS1), or MRS broth (carrier) only, were administered by intragastric gavage of a 200 µL volume once daily. The carrier and the bacterial strains were administered for five consecutive days. At day six, the mice were sacrificed, after which the spleen and mesenteric lymph nodes were removed for further analysis.

### Cell Isolation and Restimulation

After sacrificing the mice, spleens and mesenteric lymph nodes (MLN) were removed for further analysis. Single cell suspensions were made by mechanical disruption of the tissue between two glass slides in 1 mL of ice-cold RPMI containing 10% (v/v) heat inactivated fetal calf serum (FCS). Subsequently, a cell strainer was used to remove remaining clumps. The cells were washed, counted, and used for staining.

Part of the cells of the spleen and MLN were stimulated, the rest was left unstimulated. 7×10^6^ cells from the spleen and MLN were restimulated in RPMI 10% FCS containing 40 nM Phorbol 12-myristate 13-acetate (PMA) (Sigma Aldrich) and 2 nM calcium ionophore (Ca^2+^) (Sigma Aldrich). Monensin (3 µM) (Sigma Aldrich) was added to allow cytokine accumulation in the cellular cytoplasm. Cells were stimulated for four hours at 37°C, after which they washed twice in ice-cold PBS containing 2% heat inactivated FCS (FACS buffer), and used for staining.

DCs were enriched and dead cells were removed from the splenic and MLN cell suspensions. For this, 1 mL of cell suspension was loaded on 1 mL of 1-step Monocyte (Accurate Chemical and Scientific Corporation, Westbury, NY) with a density of 1.068±0.001 g/ml, and centrifuged for 30 minutes at 300×*g* at 4°C. The interface was washed twice in ice-cold FACS buffer and used for staining. After density gradient centrifugation, more than 90% of the cells were vital, which was confirmed by propidium iodide staining.

### Cell Staining

T cell stainings were performed on non-stimulated, non-enriched splenic and MLN cell suspensions. DC stainings were performed on non-stimulated, DC-enriched splenic and MLN cell suspensions. Stainings for intracellular cytokines were performed on PMA/Ca^2+^ stimulated splenic and MLN cell suspensions. The T cell cocktail contained monoclonal antibodies directed against CD3, CD4, CD8, CD25, CD69, FoxP3, or appropriate isotype controls (Table I). The DC cocktail contained monoclonal antibodies directed against CD11c, MHC II, CD19, CD80, CD86, CD103, or appropriate isotype controls (Table I). The effector T cell cocktail contained monoclonal antibodies directed against CD3, CD4, CD8, IFNγ, IL5, IL10, IL17, or appropriate isotype controls (Table 1).

**Table 1:** Antibodies.

Specificity	Clone Name	Fluorochrome	Dilution	Supplier
CD3	17A2	Pacific Blue	200×	BioLegend
CD4	RM4-5	PerCP	200×	BioLegend
CD8	53-6.7	Alexa700	50×	BioLegend
CD25	3C7	APC	100×	BioLegend
CD69	H1.2F3	PE	200×	BioLegend
FoxP3	FJK-16S	FITC	100×	eBioscience
IFNγ	XMG1.2	APC	100×	BioLegend
IL5	TRFK5	PE	25×	BioLegend
IL10	JES5-16E3	PE	25×	BioLegend
IL17a	TC11-18H10.1	APC	25×	BioLegend
Rat IgG2b	N/A	APC	100×	BioLegend
Hamster IgG	N/A	PE	200×	BioLegend
Rat IgG2a	N/A	FITC	100×	eBioscience
Rat IgG1	N/A	APC	25× or 100×	BioLegend
Rat IgG1	N/A	PE	25×	BioLegend
Rat IgG2b	N/A	PE	25×	BioLegend
CD11c	N418	APC	25×	BD Biosciences
MHC II	2G9	Biotin +streptavidin PerCP	150×	BD Biosciences
CD19	6D5	PE-Cy7	100×	BioLegend
CD80	16-10A1	PE	50×	BioLegend
CD86	PO3	Alexa700	50×	BioLegend
CD103	2E7	Pacific Blue	25×	BioLegend
Hamster IgG	N/A	PE	50×	BioLegend
Rat IgG2b	N/A	Alexa700	50×	BioLegend
Hamster IgG	N/A	Pacific Blue	25×	BioLegend

In short, 1×10^6^ cells were incubated in FACS buffer containing 10% normal mouse serum for 30 minutes to prevent non-specific antibody staining. Subsequently, the cells were incubated with a cocktail of primary antibodies for 30 minutes. The cells were fixed in FACS Lysing solution (BD Biosciences) for 30 minutes, in the dark. The tubes for intracellular cytokine staining were subsequently washed twice in 1× permeabilisation buffer (eBioscience) and incubated with the intracellular antibodies cocktails containing 2% normal rat serum in permeabilisation buffer for 30 minutes in the dark. The whole procedure was performed on ice.

### Flow Cytometry

During flow cytometry, at least 5×10^5^ cells were analyzed. Flow Cytometry was performed using the LSR II Flow Cytometer system (BD Pharmingen), using FACS Diva software. Analysis was performed using FlowJo 7.6.2 software. Lymphocytes were gated based on the expression of CD3 and CD4 or CD8. The expression of CD25, CD69, FoxP3, and cytokines was determined based on samples stained with the isotype controls. Dendritic cells were gated in the forward side scatter plot, based on size, granularity, and the expression of MHC II^+^ and CD11c^+^. CD19^+^ B-cells were excluded from analysis. The expression of CD80, CD86, and CD103 was determined based on samples stained with the isotype controls.

### Statistics

All data are expressed as the mean ± standard error of the mean (SEM). Normal distribution of the data-sets was confirmed by the Kolmogorov-Smirnov test. The one-way ANOVA, followed by a two-sided Dunnett post-hoc test was used to determine changes in immune cell populations after probiotic treatment *in vivo*. The two-sided Mann Whitney U-test was used to determine changes in cytokine release after probiotic co-incubation *in vitro.* P-values <0.05 (*) were considered statistically significant.

## Results

### Strain Specific Release of Pro- and Anti-inflammatory Cytokines by Murine DCs

**Figure 1:**
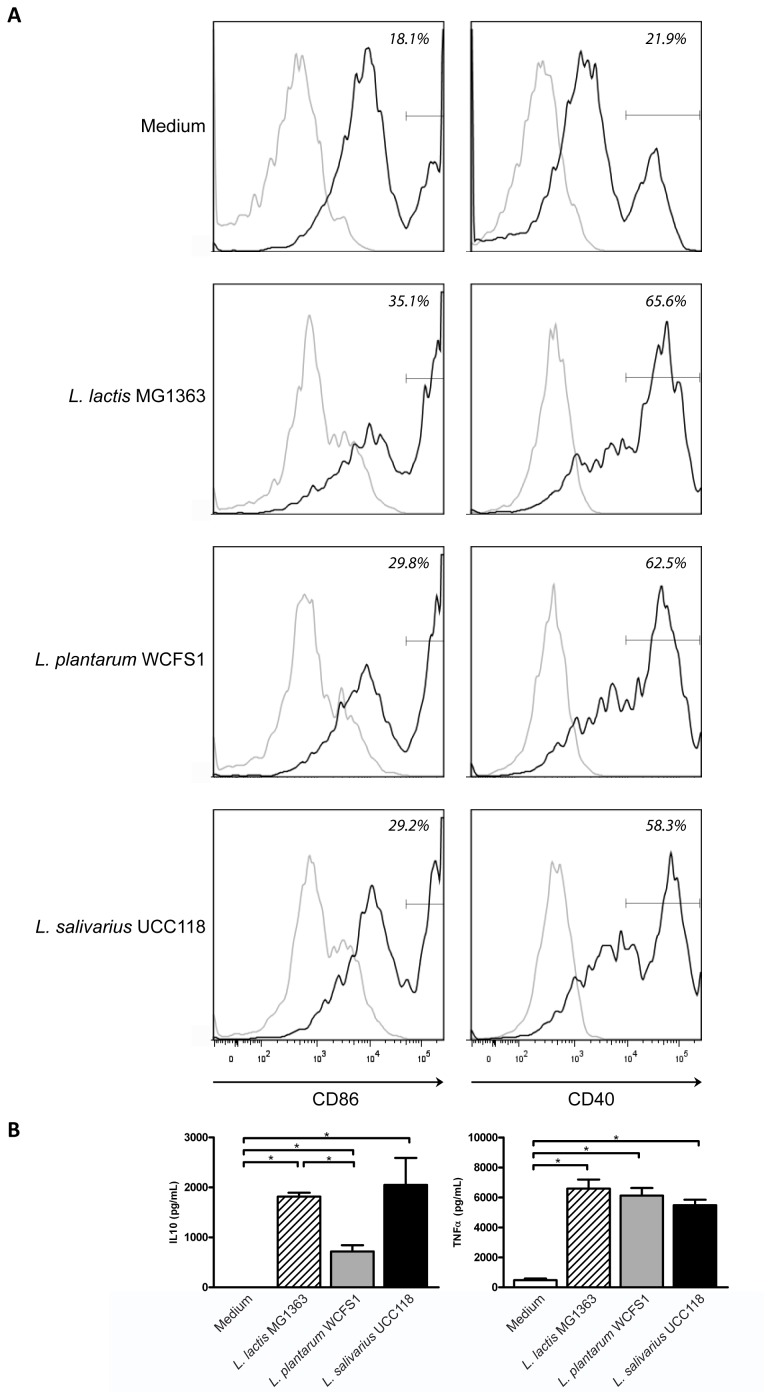
Probiotic-induced dendritic cell activation in vitro. In vitro activation of murine bone-marrow-derived dendritic cells (BM DCs) to medium, L. lactis MG1363, L. plantarum WCFS1 or L. salivarius UCC118 (N = 4). DCs were gated based on the FSC-SSC pattern and the expression of CD11c. Cell activation is demonstrated as the expression of CD86 and CD40 within the DC population (black line) as compared to DCs stained with the isotype control (grey line). Both CD86 or CD40^intermediate^ and CD86 or CD40^high^ cells (gates) were observed. A representative FACS plot is demonstrated (A). The frequency of cells in the gate is indicated. Following incubation of murine BM DCs with medium (white bars), L. lactis MG1363 (dashed bars), L. plantarum WCFS1 (grey bars), or L. salivarius UCC118 (black bars) the release of IL10 and TNFα was determined (N = 4) (B). Results are depicted as the mean ± standard error of the mean (SEM). Statistical significance was calculated using the Mann Whitney U test. * represents P-values <0.05.

To confirm the immunomodulatory potential of our probiotic strains, we first performed an *in vitro* assay. For this, bone-marrow derived (BM) dendritic cell (DC) activation and cytokine responses were determined in response to *L. plantarum* WCFS1, *L. salivarius* UCC118, or *L. lactis* MG1363 (*N = 4*). Incubation of all bacteria with BMDCs led to cellular activation, as shown by an increased frequency of BMDCS expressing high levels of the activation markers CD86 and CD40 (gate) as compared to medium stimulated BMDCs (Figure 1A). In addition to BMDC activation, the release of pro-inflammatory TNFα and anti-inflammatory IL10 was measured. All three bacterial strains induced similar levels of TNFα (Figure 1B). Similarly, all bacteria induced IL10 (Figure 1B), but the IL10 response was highest for *L. salivarius-*treated BMDCs, albeit not statistically significant (Figure 1B).

### Probiotic Treatment Increases the Frequency of Regulatory T Cells in a Strain Dependent Manner

**Figure 2:**
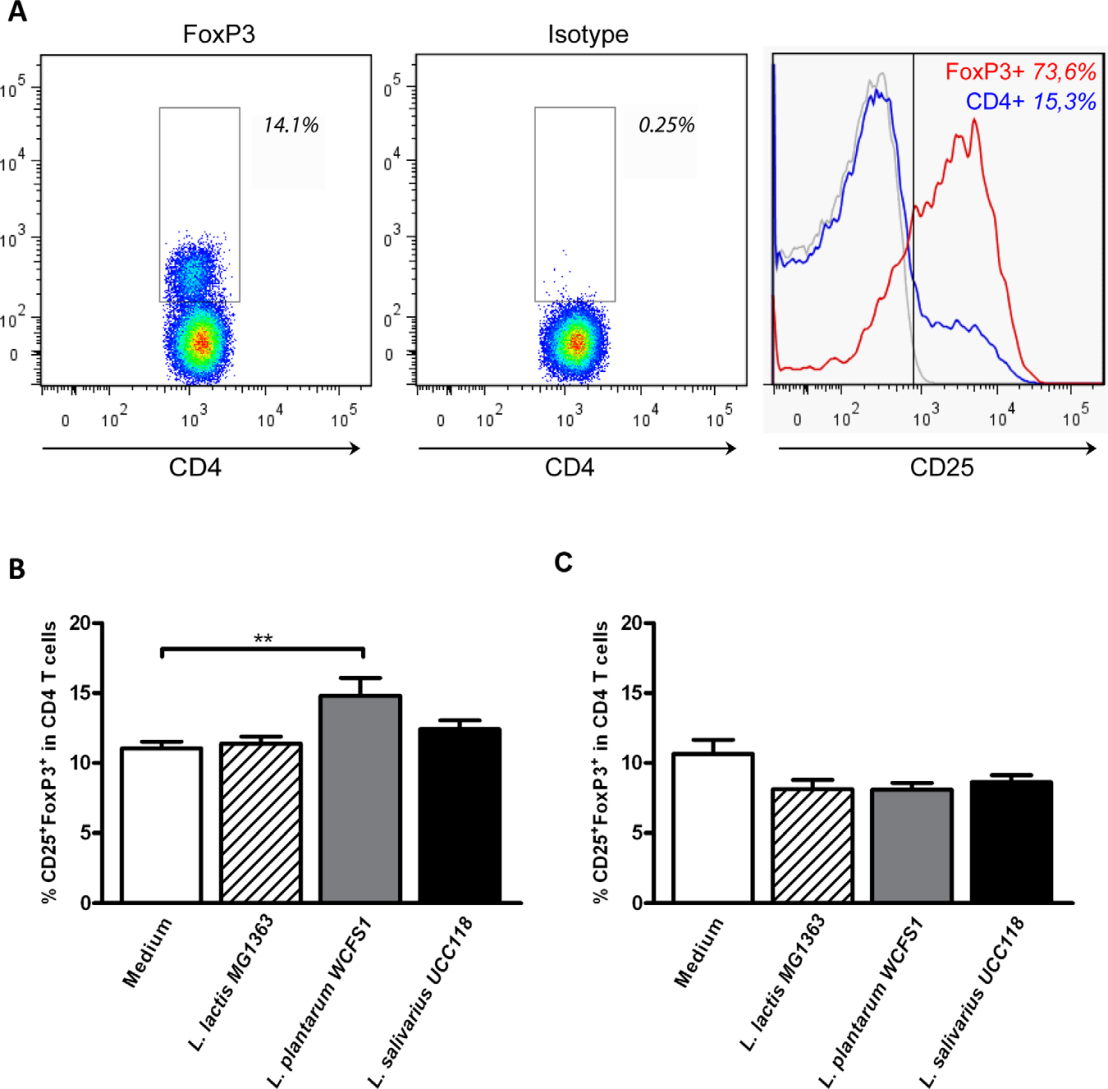
Regulatory T cells following probiotic treatment. Regulatory T cells were gated based on the expression of FoxP3 within the CD3^+^CD4^+^ T cell population. The gate was set based on staining with an isotype control. As a control, the expression of CD25 was determined within the total CD3^+^CD4^+^ T cell population (blue line) and the regulatory T cell population (red line) and compared to an isotype control (grey line). Representative FACS plots are depicted (A). Regulatory T cell frequencies in the spleen (N = 6) (B) and mesenteric lymph node (N = 6) (MLN) (C) following oral treatment with medium (white bars), L. lactis MG1363 (dashed bars), L. plantarum WCFS1 (grey bars), or L. salivarius UCC118 (black bars). Results are depicted as the mean ± standard error of the mean (SEM). Statistical significance was calculated using the One-way ANOVA followed by a two-sided Dunnet post-hoc test. * represents P-values <0.05.

Next, we evaluated the immunomodulatory properties of these strains *in vivo.* For this, we analyzed changes in the balance between different pro-inflammatory and regulatory T cell populations in the mesenteric lymph node (MLN) and spleen after consumption of the bacteria. In addition, dendritic cell (DC) frequencies and activation was determined, as DCs are important in both bacterial recognition as well as shaping local and systemic T cell responses [50]. The mice (*N = 6* per group) received *L. plantarum* WCFS1, *L. salivarius* UCC118, *L. lactis* MG1363, or culture medium alone, for 5 consecutive days. First, we focused on the frequency of regulatory T cells following probiotic treatment. Regulatory CD4^+^ T cells were defined on the basis of FoxP3 expression (Figure 2A) and consistently showed high CD25 expression as compared to the total CD4^+^ T cell population (Figure 2A).

**Figure 3:**
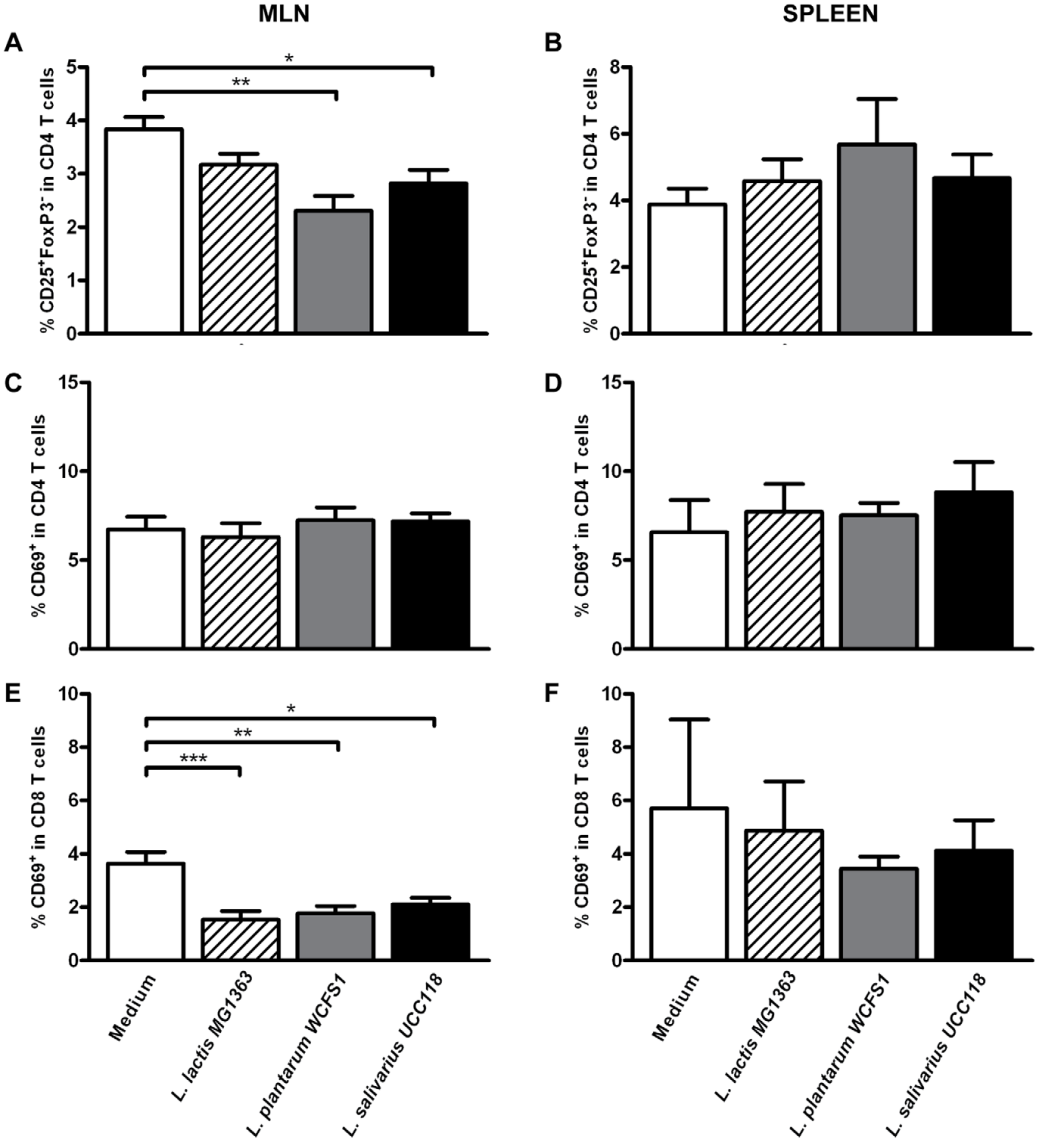
Activated CD4^+^ and CD8^+^ T cells following probiotic treatment. Activated CD4^+^ and CD8^+^ T cell frequencies in the mesenteric lymph node (N = 6) (MLN) and spleen (N = 6) following oral treatment with medium (white bars), L. lactis MG1363 (dashed bars), L. plantarum WCFS1 (grey bars), or L. salivarius UCC118 (black bars). Activated T cell frequencies are depicted as the frequency of CD25^+^FoxP3^−^ cells within CD4^+^ T cells (A&B), CD69^+^ cells within CD4^+^ T cells (C&D), and CD69^+^ cells within CD8^+^ T cells (E&F). Results are depicted as the mean ± standard error of the mean (SEM). Statistical significance was calculated using the One-way ANOVA followed by a two-sided Dunnet post-hoc test. * represents P-values <0.05, ** represents P-values <0.01.

**Figure 4:**
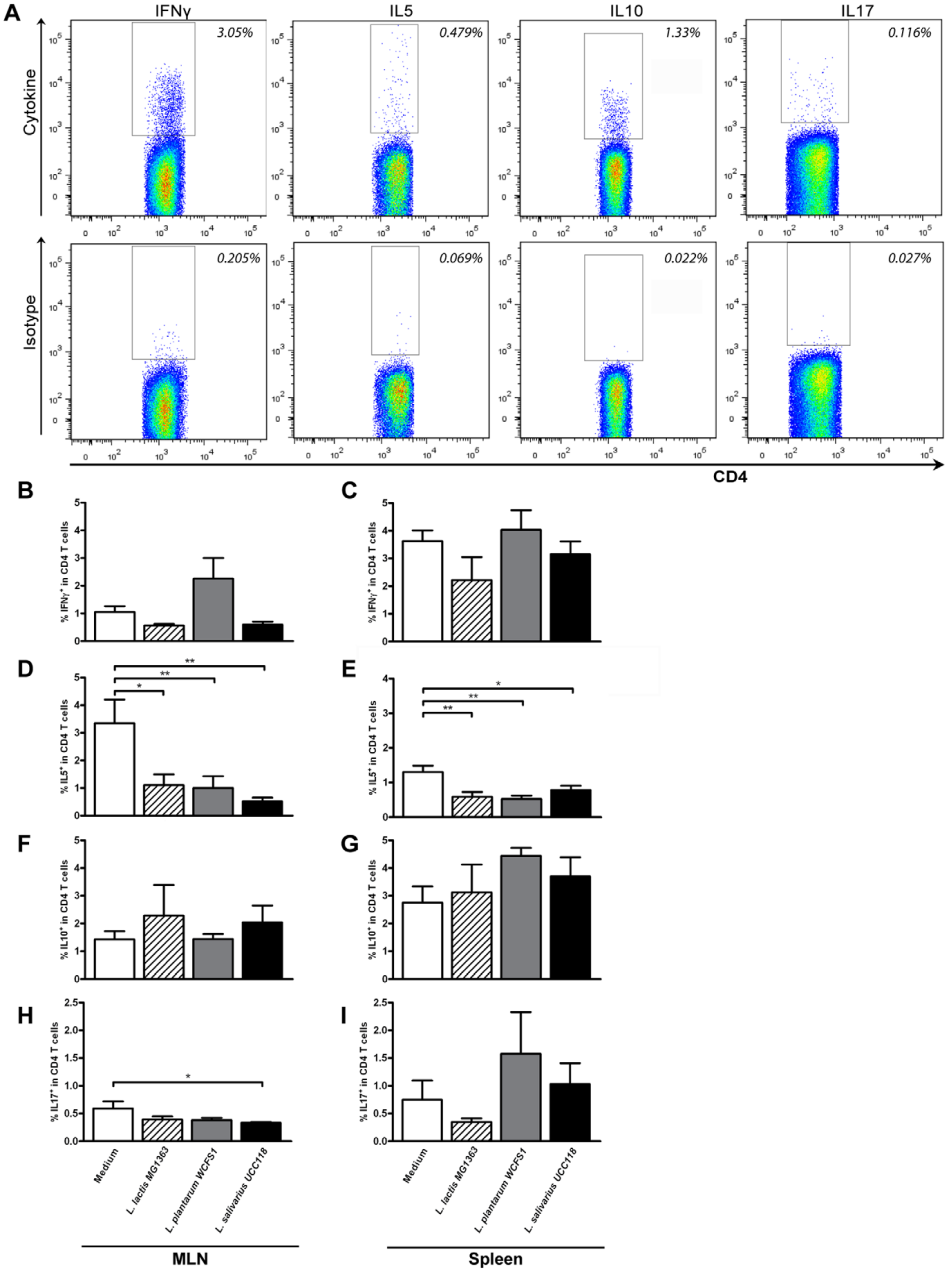
Polarized CD4^+^ T cell frequencies following probiotic treatment. Polarized CD4^+^ T cells were gated based on the expression of IFNγ, IL5, IL10, or IL17 within the CD3^+^CD4^+^ T cell population (top plots). The gate was set based on staining with an isotype control (bottom plots). Representative FACS plots are depicted (A). Polarized CD4^+^ T cell frequencies in the mesenteric lymph node (N = 6) (MLN) and spleen (N = 6) following oral treatment with medium (white bars), L. lactis MG1363 (dashed bars), L. plantarum WCFS1 (grey bars), or L. salivarius UCC118 (black bars). Polarized CD4^+^ T cell frequencies are depicted as the frequency of IFNγ^+^ cells within CD4^+^ T cells (B&C), IL5^+^ cells within CD4^+^ T cells (D&E), IL10^+^ cells within CD4^+^ T cells (F&G), and IL17^+^ cells within CD4^+^ T cells (H&I). Results are depicted as the mean ± standard error of the mean (SEM). Statistical significance was calculated using the One-way ANOVA followed by a two-sided Dunnet post-hoc test. * represents P-values <0.05, ** represents P-values <0.01.

**Figure 5:**
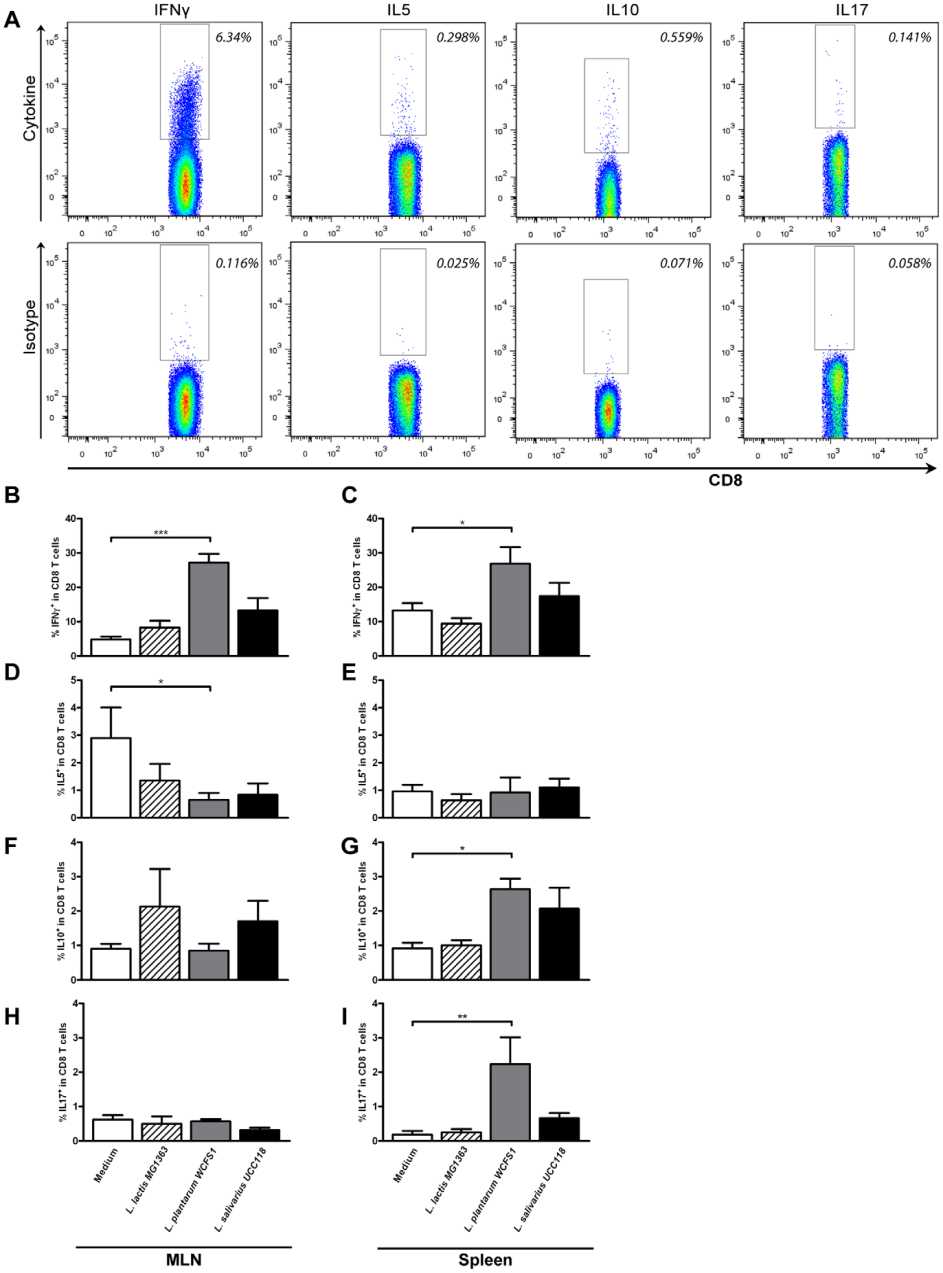
Polarized CD8^+^ T cell frequencies following probiotic treatment. Polarized CD8^+^ T cells were gated based on the expression of IFNγ, IL5, IL10, or IL17 within the CD3^+^CD8^+^ T cell population (top plots). The gate was set based on staining with an isotype control (bottom plots). Representative FACS plots are depicted (A). Polarized CD8^+^ T cell frequencies in mesenteric lymph node (N = 6) (MLN) and spleen (N = 6) following oral treatment with medium (white bars), L. lactis MG1363 (dashed bars), L. plantarum WCFS1 (grey bars), or L. salivarius UCC118 (black bars). Polarized CD8^+^ T cell frequencies are depicted as the frequency of IFNγ^+^ cells within CD8^+^ T cells (B&C), IL5^+^ cells within CD8^+^ T cells (D&E), IL10^+^ cells within CD8^+^ T cells (F&G), and IL17^+^ cells within CD8^+^ T cells (H&I). Results are depicted as the mean ± standard error of the mean (SEM). Statistical significance was calculated using the One-way ANOVA followed by a two-sided Dunnet post-hoc test. * represents P-values <0.05, ** represents P-values <0.01.

Systemic regulatory T cell frequencies were increased after probiotic treatment, in a strain-dependent manner. Only *L. plantarum*-treated mice demonstrated increased frequencies of regulatory T cells in the spleen as compared to medium treated mice (Figure 2B). This increase was not observed after treatment with *L. salivarius* or *L. lactis* (Figure 2B). In the MLN, a trend towards decreased regulatory T cell frequencies was observed after all bacterial treatments, but this never reached statistical significance [One-way ANOVA *P = 0.07* (Figure 2C)].

**Figure 6:**
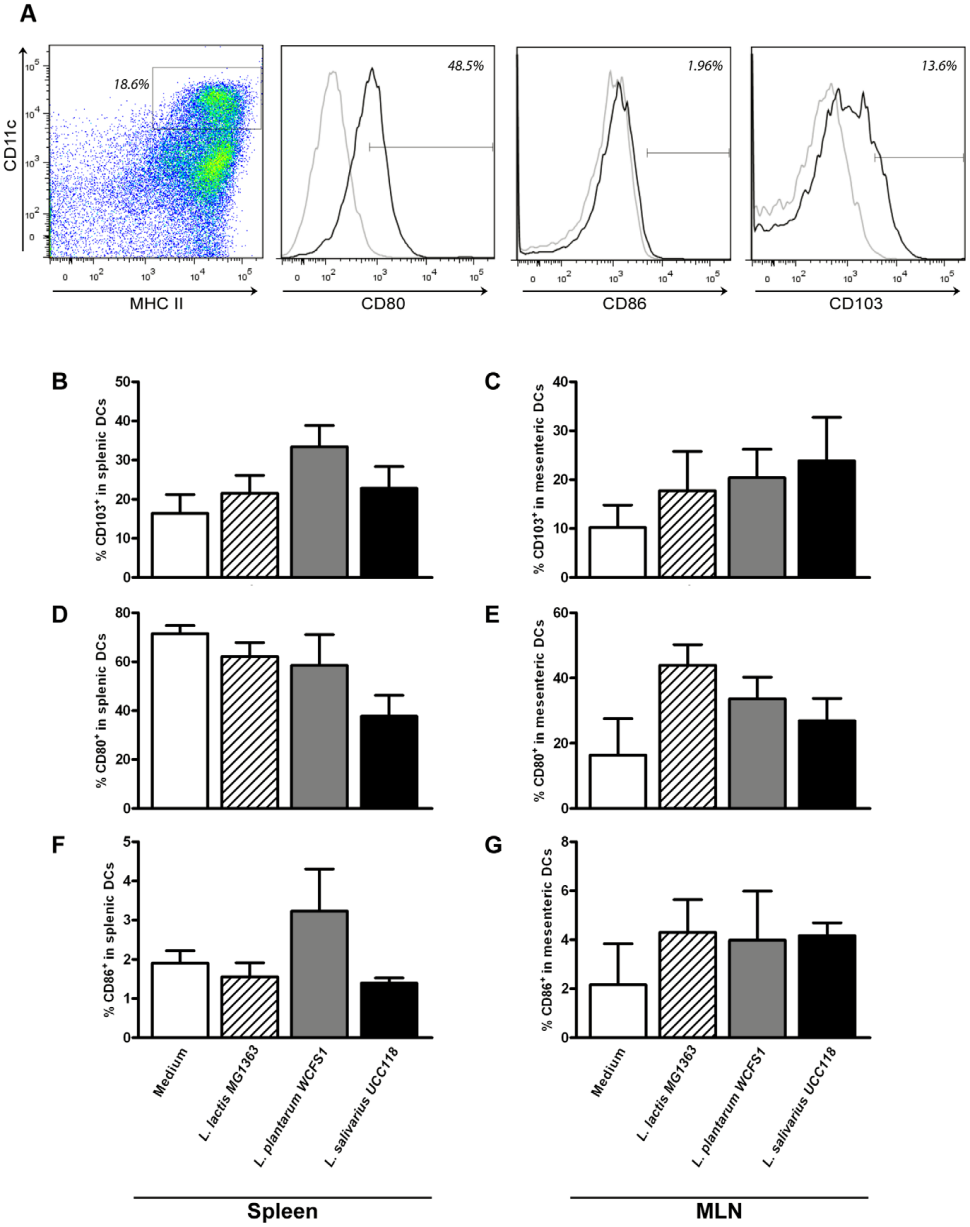
Dendritic cell frequencies and activation following probiotic treatment. Dendritic cells were gated based on the expression of CD11c and MHC II. Within the dendritic cell population the frequency of CD103^+^, CD80^+^, or CD86^+^ cells was determined (black lines). The gate were set based on staining with an isotype control (grey lines). Representative FACS plots are depicted (A). Frequency of CD103^+^ dendritic cell subsets in the spleen (N = 6) (B) or mesenteric lymph node (N = 6) (C) following oral treatment with medium (white bars), L. lactis MG1363 (dashed bars), L. plantarum WCFS1 (grey bars), or L. salivarius UCC118 (black bars). Activated dendritic cells are depicted as the frequency of CD80^+^ cells within the CD11c^+^ MHC II^+^ cells in the spleen (D) and MLN (E), or the frequency of CD86^+^ cells within the CD11c^+^ MHC II^+^ cells in the spleen (F) and MLN (G). Results are depicted as the mean ± standard error of the mean (SEM). Statistical significance was calculated using the One-way ANOVA followed by a two-sided Dunnet post-hoc test. * represents P-values <0.05.

### Probiotic Treatment Reduces the Frequency of Activated T Cells in a Strain Dependent Manner

Second, we determined the frequency of pro-inflammatory T cell populations. For this, we determined the frequency of activated T cells. Activated T cells were defined as the frequency of CD25^+^FoxP3^−^ or CD69^+^ cells within CD4^+^ T cells or CD69^+^ cells within CD8^+^ T cells.

Both probiotic strains decreased CD4^+^ and CD8^+^ T cell activation in the MLN, as demonstrated by decreased CD25^+^CD4^+^ (Figure 3A) and CD69^+^CD8^+^ (Figure 3E) frequencies following *L. plantarum* and *L. salivarius* treatment. CD69^+^CD4^+^ frequencies were not affected (Figure 3C). Treatment with *L. lactis* induced a slight reduction in the frequency of CD25^+^CD4^+^ T cells [*P = 0.06* (Figure 3A)] together with a marked reduction in the frequency of CD69^+^CD8^+^ T cells in the MLN (Figure 3E). In the spleen, T cell activation was not affected by any of the treatments (Figure 3B, 3D, 3F). These results demonstrate that probiotic treatment can modify the balance between systemic pro-inflammatory and regulatory T cell populations in a strain dependent manner.

### Probiotic Treatment Affects the Frequency of Polarized CD4^+^ T Cell Populations in a Strain Dependent Manner

In order to gain functional insight in the consequences of *L. plantarum* WCFS1, *L. salivarius* UCC118, and *L. lactis* MG1363 administration on T cell responses, we performed another series of experiments. Cells from the spleen and MLN were restimulated with PMA/Ca^2+^ *ex vivo,* after which cellular cytokine responses were determined. IFN-γ was measured as a marker for Th1 cells [51]. Th1 cells stimulate the cellular immune response, but are also involved in severe autoimmune processes [52]. IL5 is an accepted marker for the Th2 subset [51], which stimulates humoral responses, but is also involved in allergy and asthma [52]. IL10 was measured as a marker for regulatory T cells [52], which are involved in anti-inflammatory responses [52]. Finally, IL17 was measured as a marker for Th17 cells [46], [47], which are involved in responses against fungi, but also in severe autoimmune responses [52], [53]. The frequency of cytokine producing CD4^+^ T cells was determined based on appropriate isotype controls (Figure 4A).

The bacterial treatments decreased Th2 responses, which was most pronounced in the MLN, but was also observed in the spleen. Further, the effects were dependent on the probiotic strain administered. All three bacterial strains decreased the frequency of Th2 cells in the MLN and spleen 2 to 3-fold as compared to medium-treated mice (Figure 4D and 4E respectively). In addition, a trend towards increased frequencies of IL10-producing CD4^+^ T cells was observed in the spleen following *L. plantarum* treatment (Figure 4G), although it didn’t reach statistical significance. In addition to decreased Th2 frequencies, *L. salivarius* treatment also induced almost a 2-fold decrease in the frequency of Th17 cells in the MLN as compared to medium treatment (Figure 4H). Further, a trend towards a 2-fold decrease in Th1 frequencies was observed in the MLN following *L. lactis* treatment [*P = 0.06*) (Figure 4B)]. Other polarized CD4^+^ T cell frequencies were not affected by the treatments (Figure 4).

### Probiotic Treatment Affects the Frequency of Cytokine Producing CD8^+^ T Cell Populations in a Strain Dependent Manner

Similar to the CD4^+^ T cell population, CD8^+^ effector T cells can be defined on the basis of pro- and anti-inflammatory cytokine responses after *ex vivo* restimulation [54]. The frequency of cytokine producing CD8^+^ T cells was determined based on appropriate isotype controls (Figure 4B). *L. plantarum* especially, increased the responsiveness of mesenteric and splenic CD8^+^ T cells, as judged by a 3- to 4-fold increase in frequencies of IFN-γ-producing CD8^+^ T cells in the MLN (Figure 5B) and spleen (Figure 5C). Further a 4-fold increase in IL17-producing CD8^+^ T cells was observed in the spleen after *L. plantarum* treatment (Figure 5I). As found for CD4^+^ T cells, *L. plantarum* treatment decreased the frequency of IL5-producing CD8^+^ T cells in the MLN (Figure 5D), and increased the frequency of IL10-producing CD8^+^ T cells in the spleen as compared to medium treated mice (Figure 5G). *L. salivarius-*treated animals demonstrated a trend towards increased frequencies of IFN-γ-producing CD8^+^ T cells in the MLN [*P = 0.06* (Figure 5B)], albeit not statistically significant. *L. lactis* treatment did not affect the CD8^+^ T cell responsiveness (Figure 5).

### Probiotic Treatment Affects the Distribution and Activation of (Intestinal) DCs

DCs are important in both bacterial recognition as well as shaping local and systemic T cell responses [50]. Therefore, we investigated the distribution of intestinal DCs and their activation status in the spleen and MLN. DCs were defined as CD11c^+^ MHC II^+^ cells (Figure 6A). Regulatory, intestinal DCs are depicted as the frequency of CD103^+^ cells within the DC population. Also the frequency of activated DCs was determined and depicted as the frequency of CD80^+^ or CD86^+^ cells within the DC population (Figure 6A).

Probiotic treatment only modestly affected intestinal DC trafficking to the MLN and spleen. Only *L. plantarum*-treated animals demonstrated a trend towards increased CD103^+^ DC frequencies in the spleen [*P = 0.07* (Figure 6B)]. This influx of CD103^+^ DCs in the spleen was however not observed in the MLN (Figure 6B). Only *L. salivarius-*treated animals demonstrated a trend towards decreased DC activation in the spleen, as demonstrated by decreased frequencies of CD80^+^ DCs [*P = 0.06* (Figure 6D)]. The activation status of splenic and mesenteric CD103^+^ DCs was not affected by any of the treatments (not demonstrated).

## Discussion

In the present study we demonstrate that oral treatment with probiotic lactobacilli modifies the distribution of systemic (effector) T cell populations and DCs in both the MLN and the spleen. To our knowledge, this is the first report of systemic immune changes following short-term treatment with probiotic bacterial strains in healthy mice. We explored the basal immunomodulatory properties of probiotic strains in non-diseased animals and demonstrate that even a short-term period of probiotic consumption induces profound changes in cellular adaptive immune responses.

Although probiotics are generally marketed as a means to prevent disease in healthy individuals, most studies have focused on specific (intestinal) disease models [39] to demonstrate their efficacy. In such models, the immune homeostasis is often strongly perturbed and playing a role in the pathology [39], [40]. Additionally, the intestinal barrier may be compromised, altering the contact between the immune cells and the probiotic bacteria [41]. Therefore, studies in the disease state may not reflect and predict the immunomodulatory effects of probiotics in other diseases, or healthy individuals. Knowledge of how different probiotic strains can affect the immune system in the absence of disease, will gain mechanistic insights and help clarify the magnitude of their effects on the immune system, the strain dependency of these effects, their safety, and potential applications in maintaining or improving immune homeostasis. This will ultimately open up possibilities to select specific probiotics for specific immunological diseases, and as a means to prevent the development of disease [19], [55] or infection [25], [30], [31], [33] in healthy or subclinical individuals. For these reasons, we chose to study the systemic immunomodulatory properties of probiotic bacteria in healthy, non-diseased mice.

During recent years, much attention has been focused on the direct anti-inflammatory capacities of lactobacilli on tolerogenic dendritic cells (DCs) [17], the generation of regulatory T cells *in vivo* and *in vitro*, or the induction of IL10 in *in vitro* assays [17], [45], [56]–[59]. However, there are also reports that demonstrate *Lactobacillus*-induced suppression of pro-inflammatory immune responses, independent of IL10 or regulatory T cells [58], [60]–[62]. To date, it remains largely unknown in what manner lactobacilli affect the balance between of pro- and anti-inflammatory immune cell populations *in vivo*. Our data suggest that, depending on the bacterial strain administered, both processes, i.e. regulatory DC and T cell dependent and independent immunomodulation, can occur in healthy mice.

Our study clearly demonstrates that probiotic-induced immunomodulation is strain-dependent. Further, we demonstrate that strain-dependent immunomodulation is also a systemic phenomenon. *L. plantarum* WCFS1 consumption directly increased the frequency of regulatory T cells, while decreasing the responsiveness of Th2 cells and increasing the responsiveness of CD8^+^ T cells. Treatment with *L. salivarius* UCC118 did not affect the frequency of regulatory T cells; however, it decreased the responsiveness of Th2 cells. Further, *L. salivarius* treatment only modestly increased the CD8^+^ T cell responsiveness. These results have important implications for the expected health benefits exerted by the different probiotic strains.

Our results suggests that the immunomodulatory effects induced by *L. plantarum* may prove useful in the prevention or treatment of common Th2-skewed allergic diseases, such as atopic dermatitis, food allergy, and allergic asthma, and also as an adjuvant to boost the immune response to common viral infections that require the activation of CD8^+^ T cells, as for instance influenza. However, the drawback of this strain may be that it has effects on more than one immune cell subset. It may positively affect Th2-skewed allergic diseases, while at the same time it may increase the susceptibility for Th1-skewed diseases. This drawback is not seen for *L. salivarius,* which showed a clear reduction of Th2 responsiveness combined with only a modest increase in CD8^+^ T cell responsiveness. The modest effect on CD8^+^ T cell responsiveness suggests that this probiotic may be less effective in boosting responses that specifically require CD8^+^ T cell participation. Taken together, our data demonstrate that these differential probiotic immunomodulatory properties may be used to develop tailored health promoting probiotic-based strategies.

Treatment with *L. plantarum* WCFS1 was associated with profound skewing towards an immune regulatory phenotype within systemic T helper cells. These results are in line with studies that demonstrate *L. plantarum-*induced immune regulation *in vitro* [63], [64] as well as in the duodenum [38], [65]. The observed changes were accompanied by the infiltration of intestinal CD103^+^ DCs in the spleen. Since it has been demonstrated that intestinal DCs are indispensable for probiotic-induced immunomodulation *in vivo* [66] and that these cells are highly important in generating regulatory responses [67], our data suggest that systemic immune regulation is induced through *L. plantarum*-stimulated migration of regulatory CD103^+^ intestinal DCs to immunological induction sites as far as the spleen. With this study, we demonstrate that *L. plantarum* WCFS1 not only attenuates local intestinal immune responses at the site of interaction [65], but also profoundly affects the systemic immune system by skewing it to a more regulatory T helper phenotype.

*L. plantarum* WCFS1 not only skewed T helper cells towards an immune regulatory phenotype, but simultaneously increased the responsiveness of CD8^+^ T cells. This dual effect was never demonstrated for this strain before and appears to be in conflict with the observed Th hyporesponsiveness. Our observation may, however, be explained by the increased Th1/Th2 ratio caused by the reduction in Th2 frequencies. This demonstrates that *L. plantarum* not only stimulates skewing towards immune regulation, but can also directly improve the responsiveness of CD8^+^ T cells by leaving the Th1 subset unaltered. Our results corroborate previous *in vitro* findings [68], [69]. Also, several reports have indirectly demonstrated improved CD8^+^ T cell responsiveness following probiotic treatment, by demonstrating improved immune responses towards viral infections *in vivo* [28]–[32].

During recent years, many *in vitro* screening tools have been developed to predict the beneficial effect of probiotic bacteria *in vivo* [45], [62], [70], [71]. The majority of these systems focus on the secretion of only one or a few pro- and anti-inflammatory cytokines from PBMCs or DCs as a model for immunomodulation *in vivo.* In this study, we demonstrate that using this strategy potential efficacious probiotic strains may be missed. For instance *L. plantarum* has been demonstrated as a modest cytokine inducer *in vitro* [62], [70], [71], which is also apparent from the results of our *in vitro* murine dendritic cell assay. However, this strain possesses profound immunomodulatory properties *in vivo*. Further, the results from our *in vitro* assay, as well as previously published reports [57], [72] suggest that *L. salivarius* is a potent immunomodulator *in vivo*. Although *L. salivarius* had some systemic immunomodulatory effects, these were only modest and not as pronounced as the effects observed after *L. plantarum* treatment. These modest immunomodulatory effects *in vivo* may also explain the variable performance of this strain in disease models [73]–[75]. These *in vivo* disease models highly depend on Th1 reactivity [76], which, according to our data, is only marginally affected by *L. salivarius*. We therefore feel that the use of *in vitro* screening models may be valuable as a high through-put screening tool for potential probiotic strains, but the immunomodulatory properties of the bacterial strains should always be confirmed *in vivo,* or in more complex *in vitro* models.

*L. lactis* is not considered to be a strain with probiotic capacities [44], [45]. We did, however, observe immunomodulation following *L. lactis* treatment, both in our *in vitro* assay, as well as *in vivo*. Previous studies have demonstrated the importance of bacterial wall proteins for the interaction with immune cells and probiotic effects [64]. It may well be that also *L. lactis* has specific bacterial wall components that are recognized by dendritic cells in the intestine and influence DC function [77]. Much more research efforts should be employed to understand the exact interactions between bacterial wall components, specific immune cell receptors, and the consequences for the immune system, to fully understand and predict the probiotic and non-probiotic immune-modulatory effects of bacteria *in vitro* and *in vivo*. This unexpected immunomodulating potency of *L. lactis* suggests that caution should be taken in categorizing bacteria in probiotic versus non-probiotic.

Our study was not only undertaken to unravel the immediate effects of short-term administration of probiotics on cellular adaptive immune responses, but also to investigate if systemic biomarkers could be identified that would reflect the efficacy of probiotics *in vivo*. In humans, we can only access the systemic circulation to study immunomodulation and the efficacy of probiotics. As demonstrated in this study, the efficacy can effectively be assessed by measuring pro- and anti-inflammatory cytokine responses after restimulation of T cells *ex vivo.* We found clear differences, which were pronounced enough to distinguish strain dependent effects. Clear documentation of these parameters may help to understand the large differences in reported effects of different probiotic strains.

In summary, in the current study we demonstrated systemic immunomodulation following short-term oral administration of three bacterial strains in healthy mice. Although further research is necessary to investigate the implications of these immune changes for a beneficial effect in human health, our results suggest that the selection of specific probiotic strains for enforcing specific desired immune responses may be a promising strategy to improve host health.
